# Application of a novel self-assembling peptide to prevent hemorrhage after EMR, a feasibility and safety study

**DOI:** 10.1007/s00464-020-07819-7

**Published:** 2020-08-17

**Authors:** Elsa Soons, Ayla Turan, Erwin van Geenen, Peter Siersema

**Affiliations:** Department of Gastroenterology and Hepatology, Radboud University Medical Center, Geert Grooteplein Zuid 10, 6500 HB Nijmegen, The Netherlands

**Keywords:** Endoscopic mucosal resection, Topical agent, Prophylaxis, Feasibility, Safety, Delayed bleeding

## Abstract

**Background:**

A novel self-assembling peptide (SAP) can be applied to the post-endoscopic mucosal resection (EMR) defect to treat oozing bleedings. It has been suggested to stimulate early healing of damaged vessels. We hypothesized that SAP application could prevent delayed bleeding (DB) after EMR and performed a prospective cohort study to determine feasibility and safety.

**Methods:**

A total of 48 consecutive patients who underwent EMR between June 2018 and August 2019 for large lesions in the esophagus, duodenum (> 1 cm) or colorectum (> 2 cm) were treated with adjuvant SAP application. Duration and ease of SAP application were measured, as well as DB outcome.

**Results:**

The EMR defects of 48 patients were treated with SAP; 17 in the esophagus, 13 in the duodenum and 18 in the colorectum. SAP was easy to apply on the EMR defect with a median duration of 2.0 min. A dose of 3 cc was generally enough to cover a defect between 10 and 50 mm. An exploratory analysis of the prophylactic ability of SAP showed that 15.9% of patients (7/44) treated with SAP still had a DB, mostly in the duodenum (4/11). No adverse events related to gel exposure were reported.

**Conclusions:**

SAP application after EMR was found to be feasible and safe, and did not delay the procedure; however, DB was still relatively common. Future comparative studies are needed to evaluate whether SAP is able to reduce DB after EMR, particularly for lesions with an increased bleeding risk, such as in the duodenum.

**Electronic supplementary material:**

The online version of this article (10.1007/s00464-020-07819-7) contains supplementary material, which is available to authorized users.

Endoscopic mucosal resection (EMR) is a curative treatment option for dysplastic lesions and early cancers in the gastrointestinal tract. Although EMR is generally a safe procedure, adverse events still occur with delayed bleeding (DB) being the most common. DB rates after EMR have been reported between 1 and 2% in the esophagus [1–3], 0–28% in the duodenum [4, 5] and 3–12% in the colorectum [6].

Various methods have been proposed to decrease the risk of DB after EMR, such as snare tip soft coagulation of visible vessels or prophylactic clipping of the post-EMR defect. However, the benefit of these measures is still not sufficiently clear [7–10]. In the case of prophylactic clipping, the efficacy is highly dependent on achieving complete closure, which, even in experienced hands and with an adequate technique, can be difficult to achieve (e.g. when the post-EMR defect is large or located over a fold) [11]. A novel simple technique to promote hemostasis and wound regeneration is to cover the defect with a self-assembling peptide (PuraStat®, 3-D Matrix Europe, France,SAP). The matrix that is formed after application of SAP has been hypothesized to prevent DB after EMR [12]. To date, studies on SAP have mostly focused on endoscopic submucosal dissection, and less is known about the effect of SAP after EMR [12–14]. Our aim was to perform a prospective study on safety and feasibility of prophylactic SAP application after EMR. In addition, we evaluated DB rate after SAP application.

## Materials and methods

A single-center prospective cohort study was conducted at a tertiary referral center in Nijmegen, the Netherlands. Forty-eight consecutive patients who underwent EMR of the esophagus, duodenum or colorectum between June 2018 and August 2019 were treated with SAP. All EMR-procedures were performed by experienced endoscopists who each had performed > 300 previous endoscopic resections. Submucosal fluid injection in duodenal and colorectal EMR was performed with hydroxyethyl starch solution (Voluven, Fresenius Kabi, the Netherlands) colored with indigo carmine. Adrenaline was not added routinely. Standard generator settings for large polypectomies were used for all EMRs in the esophagus and colorectum (VIO 200 D; EndoCUT Q mode, Effect 3, Cut distance 1, Cut interval 6; Erbe Elektromedizin GmbH, Tübingen, Germany) and in the duodenum (VIO 200 D; EndoCUT Q mode, Effect 2, Cut distance 1, Cut interval 6). Snare tip coagulation after esophageal and colorectal EMR was performed with the settings Forced Coag, Effect 2, 60 W, and after duodenal EMR with Forced Coag, Effect 2, max 20 W. This study was approved by the Medical Ethics Review Committee of the Radboudumc, Nijmegen (reference number: 2018-4392) and was carried out in accordance with the Helsinki Declaration. The study has been registered at the Dutch Trial Register (reference number: NTR7338).

### Participants

Patients older than 18 years referred for EMR of the esophagus, duodenum or colorectum were screened. They were invited to participate when the diameter of the lesion was estimated to be ≥ 10 mm for esophageal or duodenal lesions, or ≥ 20 mm for colorectal lesions. All patients gave informed consent before initiation of the EMR-procedure.

### Materials and procedure

SAP is a transparent gel containing inactivated synthetic peptides. When these peptides come in contact with blood or tissue fluid they are pH-activated, forming a three-dimensional scaffold structure of nanofibers. This is proposed to result in a physical barrier that stops small bleedings. Furthermore, the nucleotides in the gel are supposed to promote wound regeneration [15]. The gel is applied via a dedicated catheter that is inserted through the working channel of an upper or lower endoscope (PuraStat Nozzle System type E, Top Corporation, Tokyo, Japan). After the EMR procedure, an adequate volume of SAP was applied to cover the total surface of the EMR-induced ulcer, using a 3- or 5 cc syringe. Endoscopists were instructed to desufflate air after the application of SAP. By doing this, the intestinal wall will fall back into its original state and the folds will potentially prevent migration of the gel. The decision to use other prophylactic measures (e.g. clip placement) was left to the treating physician, as current guidelines are not conclusive on this subject [16].

### Study design

All patients who were treated with SAP were prospectively followed for 30 days after the procedure. Data were collected in Castor EDC (Castor Electronic Data Capture, Ciwit BV, Amsterdam, the Netherlands), an online Electronic Data Capture platform.

### Outcomes

Primary endpoint was feasibility of SAP application, including volume used per cm^2^ of resection surface, EMR procedure time, duration of gel application and safety. Secondary outcomes included DB within 30 days post-procedure, with exclusion of patients who had undergone other prophylactic measures (e.g. clip placement), and defined as hematemesis or rectal blood loss requiring emergency room visit, blood transfusion, prolongation of hospital stay, re-hospitalization or endoscopic, radiologic or surgical intervention [17–20]. The severity of DB was described according to the ASGE guidelines [17, 19] as mild, moderate, severe or fatal. Risk of DB was evaluated according to patient related risk factors, such as location and size of the lesion, cardiovascular disease and anticoagulant use [18, 21, 22].

### Statistical analysis

Categorical data are presented as counts and percentages and compared using the *χ*^2^ test where applicable. Continuous data are reported as means ± standard deviation (SD) when normally distributed, or as median with the interquartile range (IQR) when not normally distributed and compared using the Student’s *T* test where applicable. For the interpretation of DBs, data were divided into three groups—esophageal, duodenal and colorectal lesions—because DB-risk between different locations in the gastrointestinal (GI) tract is known to vary [1–6]. All analyses were done in IBM SPSS Statistics, version 25 (SPSS Inc., Chicago, IL, USA).

## Results

### Baseline characteristics

**Fig. 1:**
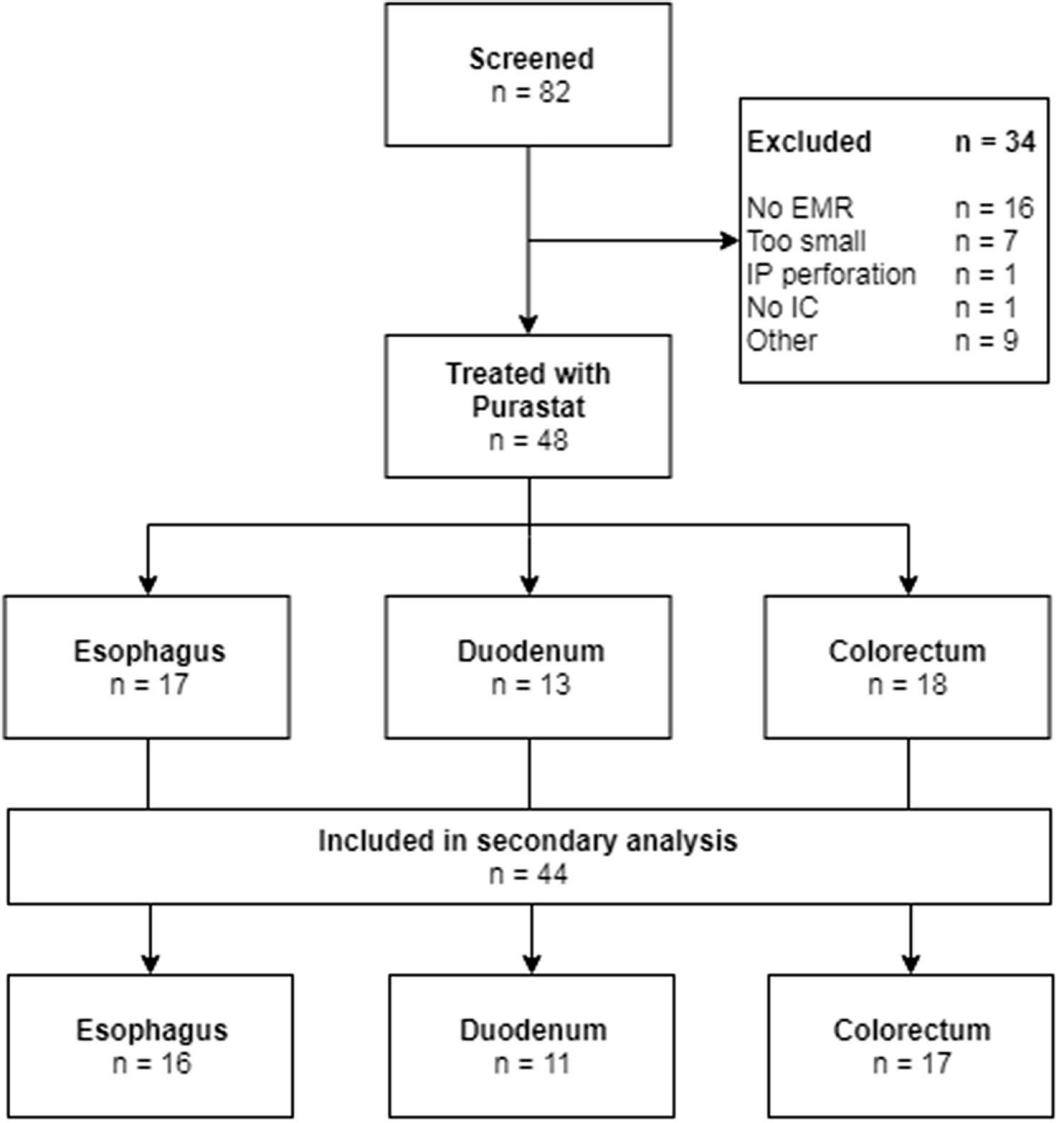
Flowchart of inclusion. *EMR* endoscopic mucosal resection, *IP* intraprocedural, *IC* informed consent

Eighty-two patients were screened for eligibility, of which 48 patients, subdivided for location in the esophagus, duodenum and colorectum, were included and treated with SAP. Four patients were excluded from the secondary analysis due to intraprocedural clip placement. The flow diagram of inclusion is presented in Fig. 1.

A total of 17, 13 and 18 patients underwent EMR of the esophagus, duodenum and colorectum, respectively. As can be seen in Table 1, the median age was 68.5 years and 60.4% were men. Most patients were graded as ASA (American Society of Anesthesiologists physical status classification) grade 2 (64.6%). A total of 22.9% of patients was smoker and 41.7% regularly used alcohol. Half of patients had cardiovascular comorbidity, mostly hypertension (20.8%). Thirty-five percent of patients used antithrombotic medication, typically a platelet inhibitor (16.7%). Antithrombotic medication was managed according to the 2016 guideline from the Dutch association of gastroenterologists and hepatologists (NVMDL), including that platelet inhibitors were generally continued and in case of double therapy clopidogrel was stopped, Vitamin K antagonists were stopped five days prior to the EMR or bridged with low-molecular-weight heparin (LMWH), and direct oral anticoagulants (DOACS) were stopped two to three days before the EMR. All antithrombotic drugs were restarted the following day, unless decided otherwise by the treating endoscopist. None of the included patients was known with portal hypertension.

**Table 1:** Baseline characteristics

	Overall *N* = 48	Esophagus *N* = 17	Duodenum *N* = 13	Colorectum *N* = 18
Age in years, median (IQR)	68.5 (55.3–73.0)	70.0 (61.0–74.5)	53.0 (46.0–71.0)	69.5 (62.0–73.3)
Sex (male), *n* (%)	29 (60.4)	12 (70.6)	8 (61.5)	9 (50.0)
BMI, mean (SD)	26.9 (3.8)	26.7 (3.6)	28.4 (3.8)	25.8 (3.9)
ASA, *n* (%)				
1	3 (6.3)	–	2 (15.4)	1 (5.6)
2	31 (64.6)	10 (58.8)	9 (69.2)	12 (66.7)
3	12 (25.0)	6 (35.3)	1 (7.7)	5 (27.8)
4	2 (4.2)	1 (5.9)	1 (7.7)	–
Smoking, *n* (%)	11 (22.9)	5 (29.4)	–	6 (33.3)
Regular alcohol intake, *n* (%)	20 (41.7)	11 (64.7)	1 (7.7)	8 (44.4)
Cardiovascular comorbidity, *n* (%)	24 (50.0)	13 (76.5)	4 (30.8)	7 (38.9)
Hypertension	10 (20.8)	6 (35.3)	1 (7.7)	3 (16.7)
Antithrombotic medication, *n* (%)				
No	31 (64.6)	10 (58.8)	11 (84.6)	10 (55.6)
Platelet inhibitors	8 (16.7)	4 (23.5)	1 (7.7)	3 (16.7)
Vit K antagonists	6 (12.5)	2 (11.8)	1 (7.7)	3 (16.7)
Combination therapy^a^	1 (2.1)	1 (5.9)	–	–
DOAC/NOAC	2 (4.2)	0	–	2 (11.1)
Portal hypertension, *n* (%)	–	–	–	–

*IQR* interquartile range, *BMI* body mass index, *SD* standard deviation, *ASA* America Society of Anesthesiologists, *Vit K antagonists* vitamin K antagonists, *DOAC/NOAC* direct oral anticoagulants/new oral anticoagulants

^a^Combination therapy defined as concurrent use of aspirin and acenocoumarin

### Primary outcome: feasibility of application

**Fig. 2:**
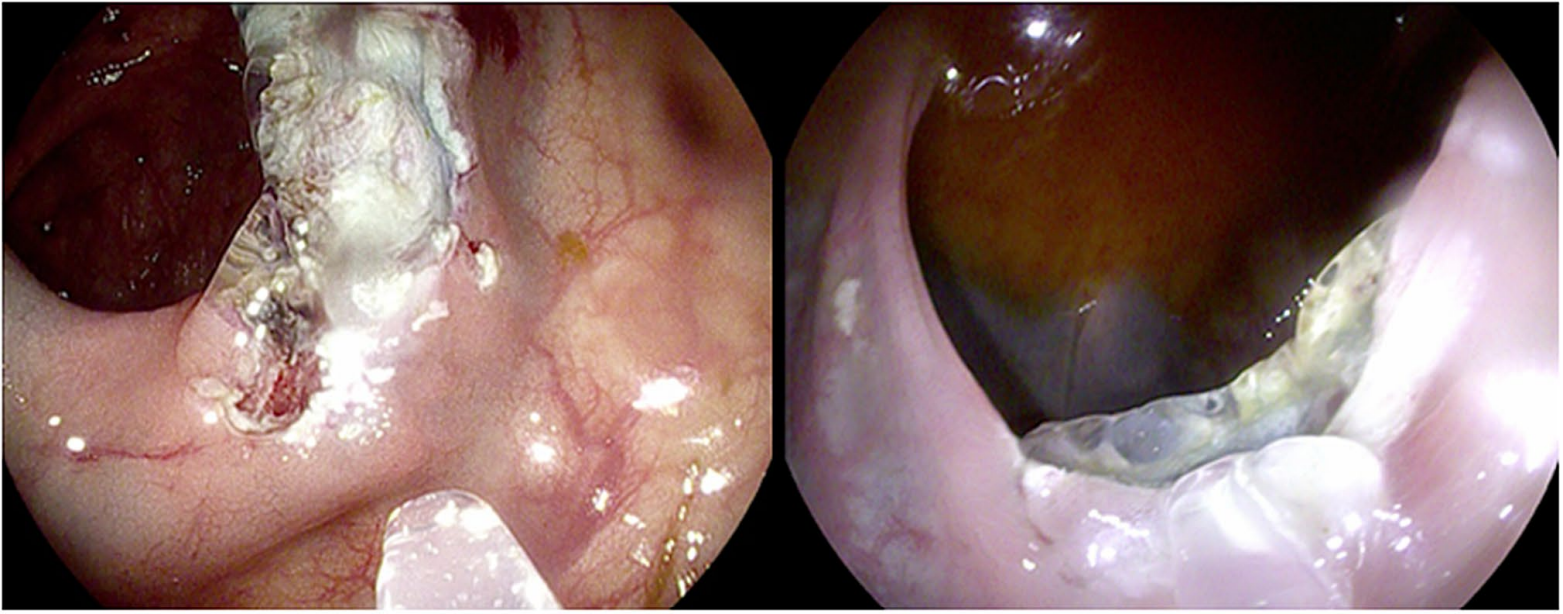
Transparent SAP applied to two separate EMR defects with a catheter

The application of the gel to the wound surface was performed systematically from the distal to the proximal side of the defect (see Video 1). SAP application was overall experienced as easy. Nonetheless, due to the transparency of the gel, it was sometimes considered difficult to confirm that the total lesion surface was completely covered (see Fig. 2). Also, the position of the EMR defect, and as a result the effect of gravity, was sometimes found to impede SAP application, which required covering some areas repeatedly (See Video 2). The median volume per cm^2^ was 0.6 cc (IQR 0.3–1.2), applied in a median time of 2.0 min (IQR 1.0–2.5) (see Table 2).

**Table 2:** Specifications of SAP use

Volume SAP in cc, median (IQR)	3.0 (3.0–5.0)
Volume SAP per cm^2^ in cc, median (IQR)	0.6 (0.3–1.2)
Time to apply SAP in minutes, median (IQR)	2.0 (1.0–2.5)
Procedure time (minutes), median (IQR)	60.0 (45.0–86.8)

*SAP* self-assembling peptide, *IQR* interquartile range

### Safety

No adverse events related to gel exposure, including allergy were reported.

### Secondary outcome: delayed bleeding

A total of 44 patients were included in the secondary outcome analysis. DB occurred in seven patients; one (6.3%) in the esophagus, four (36.4%) in the duodenum and two (11.8%) in the colorectum. Three of the patients (3/7, 42.9%) with DB used antithrombotics (see Table 3). All patients with DB underwent a piecemeal EMR.

**Table 3:** Patient related risk factors for bleeding in patients with and without DB

	EMR without DB *N* = 37	EMR with DB *N* = 7	*P* value
Age in years, median (IQR)	68.0 (57.0–72.5)	73.0 (54.0–80.0)	NS
Cardiovascular comorbidity, *n* (%)	19 (51.4)	3 (42.9)	NS
Anticoagulant use, *n* (%)			NS
No	25 (67.6)	4 (57.1)	
Antiplatelet	6 (16.2)	2 (28.6)	
Vit K antagonists	4 (10.8)	1 (14.3)	
Combination therapy^a^	1 (2.7)	–	
DOAC/NOAC	1 (2.7)	–	
Lesion location, *n* (%)			NS
Esophagus	15 (40.5)	1 (14.3)	
Duodenum	7 (18.9)	4 (57.1)	
Colorectum	15 (40.5)	2 (28.6)	
Specified location colorectum, *n* (%)			NS
Right sided	7 (46.7)	–	
Left sided	8 (53.3)	2 (100)	
Lesion size in cm^2^, median (IQR)			0.04
Esophagus	3.5 (2–6)	4 (4–4)	
Duodenum	8.5 (2–10)	8.3 (4.5–10.9)	
Colorectum	4 (2–12.5)	25.5 (NA^b^)	
Piecemeal resection, *n* (%)	32 (86.5)	7 (100)	NS
Average number of pieces	3.3	6.6	NS

*EMR* endoscopic mucosal resection, *DB* delayed bleeding, *IQR* interquartile range, *NS* not significant, *Vit K antagonists* vitamin K antagonists, *DOAC/NOAC* direct oral anticoagulants/new oral anticoagulants

^a^Combination therapy defined as concurrent use of aspirin and acenocoumarin

^b^*NA *not applicable

To assess the a priori risk of DB based on known patient related risk factors for bleeding, these risk factors were compared between patients with and without DB. The median age of patients with DB was 68 years and in those without 73 years. Although cardiovascular comorbidity, mostly hypertension, was more frequently present in the non-DB group (51.4% vs. 42.9%), platelet inhibitors were more frequently used in the DB group. Both DBs in the colorectal EMR group originated from a rectal (left sided) lesion. In addition, median size of colorectal lesions was 4 cm^2^ (2.0–12.5) in patients without DB and 25.5 cm^2^ (15–∞) in patients with DB (see Table 3).

Patients with DB presented after a median of one day (IQR 0–11) and were admitted for a median of two days (IQR 1–6), with no need for ICU admission. All patients with DB underwent additional endoscopy, with six of seven patients being treated with clip placement for active bleeding (85.7%). In one patient, clip closure was not successful because of intraprocedural clip detachment. Hemostasis of this rectal lesion was achieved after placement of two cross stich sutures and insertion of a hemostatic gelatin sponge tampon (Spongostan®). During his hospital stay, the patient received two blood transfusions. Hereafter, he recovered quickly and could be discharged within two days. In one patient with previous EMR of the duodenum, second endoscopy was required due to continued blood loss. Treatment was performed with adrenaline injection, clip placement and bipolar coagulation (Gold probe; Boston Scientific, Marlborough, MA), and no recurrent bleeding occurred. In four patients (57.1%), blood transfusion was required, with a median of two units (IQR 1.3–3.5). Most of the DBs (57.1%, see Supplementary Tables 1 and 2) were classified as moderately severe according to the ASGE lexicon for endoscopic adverse events [19].

DBs classified as mild in this cohort, were seen in patients not using antithrombotics (see Supplementary Table 3). Nonetheless, all severe and half (2/4) of the moderately severe DBs were seen in patients using antithrombotic medication prior to the EMR. One patient, who used antiplatelet therapy, stopped his medication four days prior to the EMR and resumed it 48 h after the procedure. This patient presented with a severe DB 11 days after the EMR of the esophagus. Another patient with familial adenomatous polyposis discontinued antiplatelet therapy on the day of the procedure. He was admitted the day after the EMR with a moderately severe DB. The only patient who used a vitamin K antagonist (acenocoumarin) stopped the medication three days prior to the EMR of the rectum. At the day of the procedure, the International Normalized Ratio (INR) was 1.1. Five days after the EMR, the antithrombotic medication was started again and he presented with a moderately severe DB 11 days after the EMR with an INR of 3.6.

## Discussion

This prospective pilot study confirms that prophylactic SAP application after EMR is feasible and safe and can be done in a few minutes without significantly delaying the procedure. Larger studies should be performed to determine whether SAP application is indeed effective for the prevention of DB after EMR.

We found that SAP is easy in use. A dose of 3 cc was generally sufficient to fully cover the post-EMR defect. This is comparable to the 2.56 mL that was previously reported for complete coverage of the defect after endoscopic polyp resection [14]. As the gel is transparent, a clear vision of the underlying defect plane remains possible. Nonetheless, this transparency sometimes also made it difficult to determine whether the area was fully covered. A systematic approach is therefore required, and it is advisable to desufflate during and after application for some minutes to prevent the gel from spreading over non-treated areas.

In four cases, the gel was applied in addition to clip placement (results not shown). Our experience is that the gel can easily be applied between and around the clip bases. This makes the gel suitable for potential adjuvant treatment when complete closure cannot be achieved with clips alone, which occurs in roughly one in five patients [11].

No adverse reactions related to SAP use were seen in our cohort. This includes intestinal occlusion and pancreatitis. Both were suggested in previous studies using SAP [12]. However, when one considers GI anatomy as well as the local use of the gel, these potential adverse events seem highly unlikely in relation to SAP application.

DB was seen in seven of 44 patients (15.9%), particularly in the duodenum. Although this seems relatively high, our results are in line with DB rates up to 28% for duodenal and 7–12% for colorectal lesions reported in the literature [5, 6, 11]. The DB rate of 6% after esophageal EMR seems high, compared to previously reported DB rates in the esophagus after EMR up to 2% [1–3]. Nonetheless, the esophageal DB rate in our cohort can be explained by the fact that the number of treated patients with a lesion in the esophagus was small. We expect that DB rates after EMR of both esophageal and duodenal lesions will likely be lower when more patients are included.

Nonetheless, the DB rate in our study is different from those seen in previous reports in which SAP was applied, which may in part be due to different definitions for DB and inclusion criteria, reflecting different study aims [12–14]. For example, where Subramanian et al. primarily looked into the efficacy of SAP for treatment of intraprocedural bleedings, and studied the DB rates secondary to that, we applied SAP prophylactically. Another explanation for the high DB rate could be a shift in the type of lesions referred, with more complex patients being referred to our tertiary referral center. In view of this, we also critically evaluated risk factors for DB that could potentially explain the relatively high DB rate in this cohort, reflecting a high a priori risk of DB, i.e. antithrombotic medication use, and lesion size and location. Relatively more patients with DB than without were using platelet inhibitors at the time of the EMR. Although aspirin can be continued during endoscopic interventions according to current guidelines, aspirin has been shown to increase the risk of bleeding after EMR procedures [6, 22]. Although a larger lesion size was a prerequisite for inclusion in the study, the mean size was even larger in patients developing DB (see Table 3). Various studies in the colorectum have shown that lesions ≥ 20 mm have an increased DB risk, and this risk further increases with size [21, 22]. Therefore, we believe that the high DB rate is in line with the high a priori risk of DB in these patients.

Lastly, the majority of the DBs in our cohort were observed in the duodenum (23%), which is known to be associated with an increased risk of post-EMR bleedings. Compared to the other papers on SAP, where only 15% and 10% of the EMRs were performed in the duodenum, this may also be responsible for a higher risk of DB in our cohort compared to those cohorts [12, 14].

A strength of the study is that we included both upper and lower GI lesions, which gives insight in the use of SAP at different sites in the GI tract. Furthermore, all included lesions can be considered to have a relatively high risk of DB, as we only included larger lesions [6, 20, 22]. To our knowledge, the largest reported EMR cohort with prophylactic SAP application included 21 patients [14]. In our study, more than double this number of patients were included. In addition to studying feasibility and overall safety, we also collected data on the occurrence, timing and severity of DB. We evaluated DB rates per location in the GI tract, rather than discuss all lesions as a heterogeneous group.

This study also has some limitations as it was performed in a single center, with no control group, and the number of included patients was relatively small not allowing definite conclusions about the potential for prevention of DB. Furthermore, we used a qualitative endpoint for the feasibility endpoint, i.e. whether SAP application was considered easy by the endoscopist. In this study, we were unable to determine the inter-observer variability for ease of SAP application. Nonetheless, we did quantify it in terms of application time.

In conclusion, SAP in daily practice is feasible and safe and no clear arguments against the hypothesis that SAP may prevent delayed bleeding after EMR were found. Further studies, preferably comparing SAP with other preventive measures in high-risk populations and requiring a larger sample size, are needed to show its efficacy.

## Electronic supplementary material

Below is the link to the electronic supplementary material.

Supplementary file1 (DOCX 13 kb)

Supplementary file2 (DOCX 14 kb)

Supplementary file3 (DOCX 12 kb)

Supplementary file4 (MP4 17736 kb)

Supplementary file5 (MP4 29260 kb)
