# Supplementary material for: Application of a novel self-assembling peptide to prevent hemorrhage after EMR, a feasibility and safety study
Source: Surg Endosc. 2020 Aug 17;35(7):3564–71. doi: 10.1007/s00464-020-07819-7 (PMC8195920; doi:10.1007/s00464-020-07819-7)
Supplement: Supplementary file 1 — Supplementary file1 (DOCX 13 kb) [file 464_2020_7819_MOESM1_ESM.docx]

**Supplementary files**

**Supplementary Table 1.** **Delayed bleeding characteristics**

| **Delayed bleeding** | | | 7 |
| --- | --- | --- | --- |
| Days to presentation, median (IQR) | | 1 (0 – 11) |  |
| Days of hospital admission, median (IQR) | | 2 (1 – 6) |  |
| ICU, n | 0 |  |  |
| Subsequent endoscopy | | 7 |  |
| Adrenaline injection, n (%) | 4 (57.1) |  |  |
| Clip placement, n (%) | 6 (85.7) |  |  |
| Coagulation, n (%) | 2 (28.6) |  |  |
| Hemospray, n (%) | 1 (14.3) |  |  |
| Units of blood transfusion, n | | 2 (1.3 – 3.5) |  |
| Severity | |  |  |
| Mild, n | 2 (28.6) |  |  |
| Moderate, n | 4 (57.1) |  |  |
| Severe, n | 1 (14.3) |  |  |
