# Supplementary material for: Application of a novel self-assembling peptide to prevent hemorrhage after EMR, a feasibility and safety study
Source: Surg Endosc. 2020 Aug 17;35(7):3564–71. doi: 10.1007/s00464-020-07819-7 (PMC8195920; doi:10.1007/s00464-020-07819-7)
Supplement: Supplementary file 2 — Supplementary file2 (DOCX 14 kb) [file 464_2020_7819_MOESM2_ESM.docx]

**Supplementary files**

**Supplementary Table 2.** **Delayed bleeding characteristics per location**

|  | | | Self-assembling peptide applied N = 42 |
| --- | --- | --- | --- |
| **Esophagus** | | | 1 |
| Days to presentation, n | | 11 |  |
| Days of hospital admission, n | | 16 |  |
| ICU, n | 8 |  |  |
| Intervention | |  |  |
| Endoscopy, n | 1 |  |  |
| Clip placement, n | 1 |  |  |
| Units of blood transfusion, n | | 2 |  |
| Severity | |  |  |
| Mild, n | - |  |  |
| Moderate, n | - |  |  |
| Severe, n | 1 |  |  |
| **Duodenum** | | | 4 |
| Days to presentation, median (IQR) | | 1.0 (0.25 – 1.75) |  |
| Days of hospital admission, median (IQR) | | 3.0 (1.25 – 5.5) |  |
| ICU, median (IQR) | 0.0 (0.0 – 0.0) |  |  |
| Intervention | |  |  |
| Endoscopy, n | 4 |  |  |
| Clip placement, n | 4 |  |  |
| Units of blood transfusion, median (IQR) | | 2.5 (1.0 – 4.0) |  |
| Severity | |  |  |
| Mild, n | 2 |  |  |
| Moderate, n | 2 |  |  |
| Severe, n | 0 |  |  |
| **Colon** | | | 2 |
| Days to presentation, median (IQR) | | 5.5 (0.0 – 11.0) |  |
| Days of hospital admission, median (IQR) | | 1.5 (1.0 – 2.0) |  |
| ICU, median | 0 (0.0 – 0.0) |  |  |
| Intervention | |  |  |
| Endoscopy, n | 0 |  |  |
| Clip placement, n | 1 |  |  |
| Units of blood transfusion, median (IQR) | | 2.0 (2.0-2.0) |  |
| Severity | |  |  |
| Mild, n | 0 |  |  |
| Moderate, n | 2 |  |  |
| Severe, n | 0 |  |  |
