# Supplementary material for: Application of a novel self-assembling peptide to prevent hemorrhage after EMR, a feasibility and safety study
Source: Surg Endosc. 2020 Aug 17;35(7):3564–71. doi: 10.1007/s00464-020-07819-7 (PMC8195920; doi:10.1007/s00464-020-07819-7)
Supplement: Supplementary file 3 — Supplementary file3 (DOCX 12 kb) [file 464_2020_7819_MOESM3_ESM.docx]

**Supplementary files**

**Supplementary Table 3. Severity of delayed bleeding according to antithrombotic use.**

|  | Mild | Moderate | Severe | Total |
| --- | --- | --- | --- | --- |
| Yes, antiplatelet | 0 | 1 | 1 | 2 |
| Yes, vitamin K antagonist | 0 | 1 | 0 | 1 |
| No | 2 | 2 | 0 | 4 |
| Total | 2 | 4 | 1 | 7 |
